# The impact of a structured weight-loss treatment on physical fitness in patients with psoriatic arthritis and obesity compared to matched controls: a prospective interventional study

**DOI:** 10.1007/s10067-022-06164-5

**Published:** 2022-06-01

**Authors:** Annelie Bilberg, Ingrid Larsson, Sofia Björkman, Björn Eliasson, Eva Klingberg

**Affiliations:** 1 Institute of Neuroscience and Physiology, Section of Health and Rehabilitation, Physiotherapy, Sahlgrenska Academy, University of Gothenburg, Gothenburg, Sweden; 2 Department of Gastroenterology and Hepatology, Sahlgrenska University Hospital, Gothenburg, Institute of Medicine, Sahlgrenska Academy, University of Gothenburg, Gothenburg, Sweden; 3 Department of Gastroenterology and Hepatology, Sahlgrenska University Hospital, Gothenburg, Sweden; 4 Department of Rheumatology and Inflammation Research, Sahlgrenska Academy, University of Gothenburg, Gothenburg, Sweden

**Keywords:** Physical fitness, Psoriatic arthritis, Obesity, Very low energy diet treatment

## Abstract

**Objectives:**

To evaluate the effects of weight loss treatment on physical fitness in patients with psoriatic arthritis (PsA) and obesity compared to matched controls.

**Methods:**

In total, 46 patients with PsA (CASPAR) and BMI ≥ 33 kg/m^2^ and 52 obese persons were included in this 12-month prospective open intervention study with a very low energy diet (640 kcal/day), followed by structured reintroduction of an energy-restricted diet and brief support for physical activity. The primary outcome was muscle strength assessed with hand-grip strength (Grippit) and leg muscle strength (timed stand test). Secondary outcomes were cardiorespiratory fitness, body composition, and physical functioning (SF-36PCS). Outcomes were assessed at baseline, 6 (M6), and 12 months (M12). Nonparametric statistics were used.

**Results:**

Median weight reduction at M6 was 18.9 kg in patients and 23.0 kg in controls, (*p* = 0.546). At M12, patients’ median weight loss from baseline was 16.1 kg, corresponding with significant loss of total fat mass (− 30.1%), and lean mass (total − 7.0%, arm − 13.7%, and leg − 6.0%). Leg muscle strength improved in patients and controls at M6 (*p* < 0.001) and remained improved at M12 (*p* < 0.01), while hand-grip strength was unchanged in both groups. Cardiorespiratory fitness increased in controls at M6 (*p* = 0.018) and M12 (*p* = 0.028) but not in patients. Physical functioning improved in both groups at M6 (*p* < 0.001) and remained improved at M12 (*p* = 0.008) and (*p* < 0.01), respectively.

**Conclusion:**

The intervention resulted in positive effects on body weight and total body fat. Despite reduced lean body mass, the muscle strength did not deteriorate in patients with PsA and controls.

**Trial registration:**

ClinicalTrials.gov identifier: NCT02917434, registered on September 21, 2016-retrospectively registered.

**Table:** 

**Key Points** *• Patients with PsA and obesity can benefit from weight loss treatment without the risk of deterioration in muscle strength and cardiorespiratory fitness.* *• Muscle strength and cardiorespiratory fitness were below suggested normative values for the majority of the patients at all time points, implying that more structured exercise strategies might be warranted to counteract physical fitness deficiencies in patients with PsA undergoing weight loss treatment.*	

## Introduction

Psoriatic arthritis (PsA) is a chronic inflammatory rheumatic disease involving the peripheral joints, spine, and sacroiliac joints. Clinically, patients with PsA display arthritis, spondylitis, enthesitis, and dactylitis leading to pain, stiffness, and physical limitations [1]. They also report higher activity limitations and decreased health-related quality of life compared to healthy controls [2]. Obesity and PsA often coexist [3, 4]. Approximately 30–45% of the adult patients with PsA are obese, i.e., body mass index BMI ≥ 30 kg/m^2^ [3]. Moreover, obesity in PsA is associated with increased disease activity and poorer treatment response [5]. Besides obesity, patients have an increased risk of different comorbidities [6], including a substantially heightened risk of cardiovascular diseases (CVD) [7].

Obesity is associated with a sedentary lifestyle which adds to the risk of CVD [8] and CVD mortality [9]. Long duration of sedentarism and excess weight is suggested to adversely affect muscle strength and cardiorespiratory fitness in obese individuals [10]. In addition to obesity, the consequences of rheumatic disease such as joint stiffness and pain may further increase the risk of physical inactivity and a sedentary lifestyle in patients with PsA and obesity. A first-line treatment approach to counteract obesity is dietary energy restriction along with lifestyle changes and increased physical activity [11]. In severe obesity, BMI ≥ 35.0 kg/m^2^, a rigorous energy restriction is needed for optimal weight loss, and weight loss treatment with a very low energy diet (VLED) (< 800 kcal/day) is effective and recommended method in clinical use [12]. A side effect with a large weight loss, ≥ 10%, is, however, a concomitant reduction of muscle mass, which can negatively affect physical fitness [13, 14]. Physical fitness has been defined by Caspersen et al. as “a set of attributes that people have or achieve that relates to the ability to perform physical activity” [15]. The health-related components of physical fitness include cardiorespiratory fitness, muscle strength, muscle function, body composition, and flexibility [15]. Physical fitness is associated with maintaining physical independence over time [16]. Compared to healthy controls, cardiorespiratory fitness [17, 18] and muscle function [18, 19] are lower in patients with inflammatory rheumatic diseases. The knowledge of physical fitness in patients with PsA and obesity is, however, scarce.

We have previously shown that weight loss with VLED improves disease activity in patients with PsA and obesity [20, 21]. How a large weight loss affects physical fitness in PsA has to our knowledge not been examined previously. We, therefore, aimed to evaluate the effects of a structured dietary weight loss treatment program of 12 months on physical fitness, as objectively measured by muscle strength, cardiorespiratory fitness, body compositions, and self-reported physical functioning in patients with PsA and obesity compared to matched controls undergoing the same treatment.

## Materials and methods

This is a secondary analysis of a prospective open intervention study evaluating the effects of a structured dietary weight loss program on disease activity in patients with PsA and obesity [20].

### Patient group selection

Patients from the rheumatology units at Sahlgrenska University hospital in Gothenburg, Borås hospital and Alingsås hospital in Sweden and fulfilling the classification criteria for psoriatic arthritis (CASPAR) [22], body mass index (BMI) ≥ 33 kg/m^2^, age 20–75 years and no change in treatment with conventional synthetic and/or biologic disease-modifying anti-rheumatic drugs (cs and/or bDMARDs) 3 months prior to recruitment were eligible for inclusion. Exclusion criteria were pregnancy, porphyria, epilepsy, type 1 diabetes, severe heart, kidney or catabolic disease, binge eating disorders, treatment with warfarin, lithium or phenytoin, mental imbalance affecting participation, heart infarction, stroke, major surgery, or trauma during the last 3 months, and being treated for cancer during the last 5 years.

### Control group selection

A control group of obese persons planned for treatment with VLED, matched at group level for sex, age, and body weight, was consecutively recruited from the Regional Obesity Centre at Sahlgrenska University Hospital. Exclusion criteria were the same as for the PsA group, with an additional exclusion criterion of any rheumatic disease and psoriasis.

All the participants in the study gave their written informed consent. The study was approved by the Regional Ethics Committee in Gothenburg and carried out in accordance with the Helsinki declaration. The trial was registered in ClinicalTrials.gov identifier: NCT02917434.

### Intervention

All patients with PsA and controls in the study received weight-loss treatment with VLED at the Regional Obesity Centre at Sahlgrenska University Hospital, within a framework of medical follow-up, dietary energy restriction, and support for 12 months [20]. The well-being of the participants was monitored at each monthly visit to the obesity clinic by a nutritionist and a nurse. A physician and specialist in internal medicine were also available to monitor medical treatments, laboratory results, and possible adverse events.

The VLED treatment consisted of four portions of powder dissolved in cold or hot water consumed as shakes or soups, providing a total daily intake of 640 kcal. (Cambridge Weight Plan Limited, Corby, UK). Depending on baseline BMI, < 40 or ≥ 40 kg/m^2^, the strict VLED treatment was maintained during 12 or 16 weeks, i.e., participants with BMI < 40 kg/m^2^ received VLED for 12 weeks, and participants with BMI ≥ 40 kg/m^2^ received VLED for 16 weeks. After the VLED period, food was gradually reintroduced during a period of 12 weeks, and each participant was given personal dietary advice for further weight loss. Additionally, the participants received individual counseling by a physiotherapist for physical activity in their own environment. Including information on the health benefits of the general recommendation for physical activity, ≥ 150 min of moderately intense weekly activity, and reduced amount of sedentary time. Moreover, based on the participant’s physical capacity, medical status, current physical activity level, preferences, and possible barriers, alternatives for physical activity were thereafter discussed and planned in cooperation with the participant. During the 6- and 12-months visits, the counseling was repeated. The physical activities preferred by most participants were brisk walking, bicycling, aqua aerobic, swimming, and yoga. To reduce the risk of musculoskeletal injuries and cardiovascular complications, a gradual increase in time or intensity for physical activity was strictly recommended.

### Assessments and outcomes

Background data and outcomes, comprising medical examination, blood sample, anthropometric measures, self-reported questionnaires, and three performance-based tests (the Grippit, the timed stand test, and the Åstrand’s submaximal ergometic test) were assessed at baseline, and at 6 and 12 months. Body composition with dual-energy x-ray absorptiometry (DXA) was assessed at baseline and at 12 months.

Muscle strength was selected as the primary outcome since a large weight loss has been found to affect muscle mass negatively [14]. Cardiorespiratory fitness, body composition, and physical functioning were selected for secondary outcomes, as a large weight loss was thought to impact these variables.

## Primary outcomes

Hand-grip strength was assessed with a digital electronic dynamometer, the Grippit (AB Detektor Gothenburg, Sweden), which measures grip force in Newton (N) [23]. The peak grip force was assessed, where the best performance out of three trials was recorded. Muscle strength of the lower extremities was assessed with the timed stand test *(TST)* [24]. The time needed to stand up 10 times from a standard chair was recorded.

## Secondary outcomes

Cardiorespiratory fitness was performed on a cycle ergometer (Monark Ergometer 839 E, Monark Exercise AB) using the Åstrand’s submaximal ergometic test [25]. The VO_2_ max was estimated using the Åstrand-rhyming nomogram [25] based on mean HR at steady state and the mechanical load and corrected for age. The participant was instructed how to perform the test and was allowed to get into a correct pedal frequency of 60 revolutions per minute before the test started. All performance-based tests are validated for patients with inflammatory rheumatic diseases [23, 26, 27]. Body composition was evaluated by a DXA scanner (DPX-IQ densitometer; Lunar, Madison, WI, USA). For scanning, the participants were placed in a supine position with their arms held against the sides of the body. Lunar software was used to analyze the scans, yielding estimates of body fat and lean mass (in kilograms). The participant’s body weight, height, and waist circumference were measured, and BMI was calculated. Physical functioning was assessed with the physical component scale (PCS) of the short form health survey (SF-36), a generic instrument assessing health-related quality of life [28].

## Additional outcomes

Activity-induced pain during hand-grip strength was assessed with a visual analog scale (VAS 0–100). Leisure-time physical activity (PA) was assessed with the Saltin–Grimby physical activity level scale (SGPALS), a questionnaire grading PA in four levels: ^1^sedentary activities; being almost completely inactive, ^2^light PA; some PA during at least 4 h/week, ^3^moderate PA; regular PA and training for at least 2–3 h/week, ^4^vigorous PA; regular hard physical training for competitive sports several times per week [29]. Disease activity in patients with PsA was assessed with the disease activity score of 28 joints (DAS28) based on CRP [30, 31] and the disease activity in psoriatic arthritis (DAPSA) score [32]. Joints were examined with 66/68 swollen/tender joints count. Enthesitis was assessed with the LEEDS index [31]. General health perception, global pain, and global fatigue were assessed with visual analog scales (VAS 0–100), where a higher score indicates worse health and symptoms.

### Statistical analyses

Descriptive statistics for continuous data are presented as the median and interquartile range (IQR) and data for categorical variables as number (percentage). For comparison between patients and the control group, the Mann–Whitney *U* test was used for continuous variables, the Pearson chi-square test, or Fisher’s exact test for categorically variables. The Wilcoxon signed-rank test was used for comparison of continuous related samples and Mc Nemar’s test to compare categorical related samples. All significance tests were two-sided and conducted at the 5% significance level. Only the participants who attended the 6- and 12-month visits, respectively, were included in the statistical analyses. Statistical analyses were made using SPSS Statistics version 25 (IBM, Chicago, USA). The comparison of outcome variables between PsA and controls was also adjusted for age, sex, and BMI. The adjustment was performed with multivariable logistic regression with a group (PsA/controls) as the dependent variable, outcome variable Δ Grippit, Δ TST, Δ O2/l (12 months-baseline) as the main independent variable, and possible confounders as additional independent variables.

## Results

**Fig. 1:**
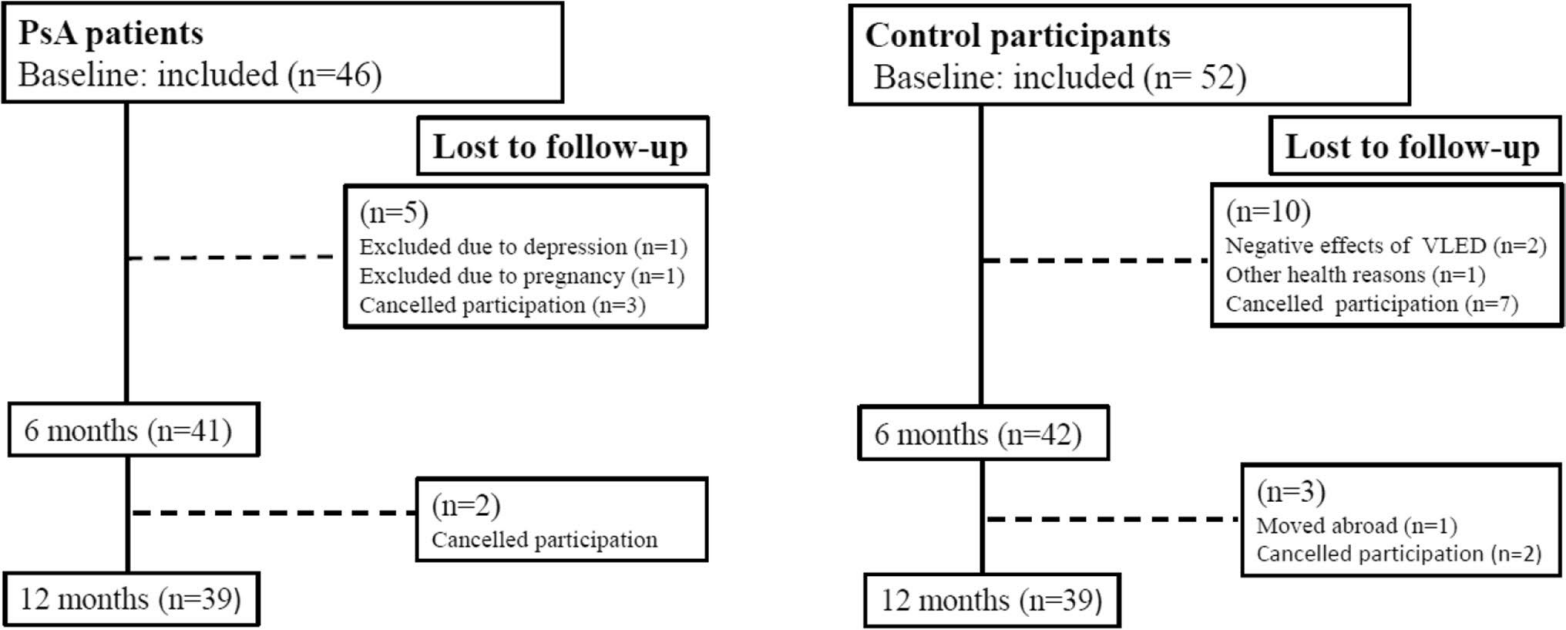
Flow chart for the study from baseline to 12 months showing participation and participants lost to follow-up

In total, 46 patients with PsA and 52 matched control participants were included in the study. Five patients (11%) and 10 controls (19%) withdrew during the VLED treatment leaving 41 patients and 42 controls to complete the 6 months visit. A total of 39 patients and 39 controls completed the 12 months visit. The sex, age, and BMI distributions were not significantly different among the participants that withdrew or completed the study. Included participants and those lost to follow-up are shown in Fig. 1. The demographics and clinical characteristics of the participants are shown in Tables 1 and 2.

**Table 1:** Characteristics of the study population at baseline and study start

	PsA group *n* = *41*	Control group *n* = *42*	*P-value*
Sex			0.347
Women *n* (%)	26 (63.4)	31 (73.8%)	
Men *n* (%)	15 (36.6)	11 (26.2%)	
Age, years	54.0 (48.5; 62.0)	54.5 (46.2;60.0)	0.450
Body measurements			
Body height, cm	168.0 (161.5; 176.8)	165.5 (162.0; 171.5)	0.171
Body weight, kg	106.3 (95.8; 113.6)	107.0 (97.0; 122.2)	0.313
Pharmacological treatment			
Anti-hypertensives, *n* (%)	18 (43.9)	18 (42.9)	0.923
Lipid lowering therapy, *n* (%)	6 (14.6)	11 (26.2)	0.192
Oral anti-diabetics, *n* (%)	1 (2.4)	4 (9.5)	0.360

For categorical variables, number (percentage) is presented. For continuous variables, median (IQR) per participant is presented

**Table 2:** Disease characteristics of the patients with PsA at baseline

Variable	Patients *n* = *41*
PsA peripheral arthritis, *n* (%)	35 (85.4)
PsA axial disease, *n* (%)	2 (4.9)
PsA peripheral and axial combination, *n* (%)	4 (9.7)
Disease duration, years	17 (11; 27)
DAPSA, score	15.3 (6.6; 29.0)
DAS28-CRP, score	2.9 (2.1; 3.7)
CRP, mg/l	4.0 (2.0; 8.5)
Tender joints 68, count	4.0 (0; 4.0)
Swollen joints 66, count	0.0 (0; 1.0)
LEEDS-index, count	1.0 (0; 4.0)
General health VAS, 0–100	34.0 (19.0; 61.0)
Global pain VAS, 0–100	30.0 (18.5; 62.5)
Global fatigue VAS, 0–100	25.0 (8.0; 44.0)
Pharmacological treatment	
NSAIDs, *n* (%)	27 (65.9)
TNFi in monotherapy, *n* (%)	4 (9.8)
TNFi + csDMARD, *n* (%)	11 (26.8)
Ustekinumab in monotherapy *n* (%)	1 (2.4)
csDMARD in monotherapy, *n* (%)	19 (46.3)
Prednisolone, *n* (%)	2 (4.9)

For categorical variables, number (percentage) is presented. For continuous variables, median (IQR) per participant is presented. *VAS*, visual analog scale; *bDMARD*, biologic disease modifying anti-rheumatic drug; *csDMARD*, conventional synthetic disease modifying anti-rheumatic drug; *NSAID*, nonsteroidal anti-inflammatory drug; *TNFi*, tumor necrosis factor inhibitor

### Body composition at baseline

A significant group difference was found for total body fat mass (*p* = 0.041) and BMI (*p* =  < 0.001), where the controls had more body fat mass and higher median BMI. No significant group differences were found for total body lean mass (*p* = 0.905), lean arm mass (*p* = 0.542), and lean leg mass (*p* = 0.489) (Table 3).

**Table 3:** Body compositions at baseline and 12 months for the patients with PsA and the controls

	PsA group		Within-group difference *p-value*	Control group		Within-group difference *p-value*	Between-group difference *p-value*	
	BL (*n* = 41)	M12 (*n* = 39)	BL-M12	BL (*n* = 42)	M12 (*n* = 39)	BL-M12	BL	M12
Body weight, kg	106.3 (95.8; 113.6)	87.5 (80.6; 95.5)	** < 0.001**	107.0 (97.0; 122.2)	87.6 (78.2; 97.5)	** < 0.001**	0.313	0.730
BMI, kg/m^2^	35.2 (34.1; 38.1)	30.5 (28.0; 32.9)	** < 0.001**	38.5 (36.9; 41.7)	32.6 (30.3; 34.8)	** < 0.001**	** < 0.001**	**0.021**
WC, cm	116.0 (112.0; 122.0)	97.5 (90.0; 105.0)	** < 0.001**	117.0 (107.0; 126.5)	100.0 (92.0; 109.0)	** < 0.001**	0.680	0.572
Tot fat mass, kg	48.5 (41.7; 56.7)	33.9 (25.9; 40.5)	** < 0.001**	50.7 (46.2; 59.2)	36.8 (27.8; 39.5)	** < 0.001**	**0.041**	0.880
Tot lean mass, kg	51.9 (45.9; 61.8)	48.3 (43.6; 58.3)	** < 0.001**	49.7 (46.9; 58.6)	45.6 (44.5; 57.9)	** < 0.001**	0.905	0.880
Lean mass arm, kg	2.8 (2.5; 3.9)	2.5 (2.1; 3.4)	** < 0.001**	2.6 (2.4; 3.3)	2.6 (2.2; 3.4)	** < 0.001**	0.542	0.651
Lean mass leg, kg	8.9 (8.0; 10.9)	8.4 (7.2; 9.8)	** < 0.001**	9.1 (8.5; 10.4)	8.1 (7.7; 9.8)	** < 0.001**	0.489	0.930

For continuous variables, median (IQR) per participant is presented. *BMI*, body mass index; *WC*, waist circumference. Missing values at 12 months: control group DXA (*n* = 19)

### Body composition 6 months and 12 months

No significant group differences were found for total weight loss at 6 months (*p* = 0.313) and 12 months (*p* = 0.730). The weight reduction at 6 months visit was a median of 18.9 kg (18.6%); a range of 8.5 to 40.2 kg in the PsA group and 23.0 kg (21.2%); from range − 2 to 44.1 kg in the control group. At 12 months, the median weight loss from baseline in the PsA group was 16.1 kg (16.0%); from range 2.7 to 37.1 kg, with a significant total fat loss of 30.1%, total lean mass loss of 7.0%, lean arm mass loss of 13.7%, and a lean leg mass loss of 6.0% (all *p* < 0.001). A similar weight loss was observed in the control group at 12-months, 16.6 kg (15.7%); range − 4.5 to 46.5 kg, with a significant total fat loss of 27.4%, total lean mass loss of 8.3%, lean arm mass loss of 2.4%, and a lean leg mass loss of 8.6% (all *p* < 0.001) (Table 3). Of the 39 patients and 39 controls who attended the 12-month visit, 39, respectively, 20 performed the DXA assessment.

### Muscle strength, cardiorespiratory fitness, and physical functioning at baseline

The majority of the study population reported right-hand dominance, 95.6% of the patients and 94.2% of the controls. No significant group difference was found regarding right-hand dominance. Hence, only the right hand is presented. The PsA group had significantly lower median peak hand-grip strength (*p* = 0.018) and self-reported median physical functioning (SF-36PCS) (*p* = 0.019) compared to the control group at baseline, while the median activity-induced hand pain was significantly higher in the PsA group (*p* < 0.001). No significant baseline differences were found for median muscle strength in the lower extremities (TST) (*p* = 0.240) and median cardiorespiratory fitness (O_2_ ml/kg × min) (*p* = 0.167), (O_2_ l/min) (*p* = 0.707), between the PsA group and the control group (Table 4). Twenty-eight of the patients and 35 of the controls underwent the test for cardiorespiratory fitness. Reasons for not performing the test were pain from the lower extremities and pharmacological treatment with beta-blockers.

**Table 4:** Muscle strength, hand pain, cardiorespiratory fitness, physical functioning, physical activity at baseline, and 6 months and 12 months in the patient and control group

	PsA group			Within-group difference *p-value*		Control group			Within-group difference *p-value*		Between-group difference *p-value*		
	BL (*n* = 41)	M6 (*n* = 41)	M12 (*n* = 39)	BL-M6	BL-M12	BL (*n* = 42)	M6 (*n* = 42)	M12 (*n* = 39)	BL-M6	BL-M12	BL	M6	M12
Outcomes													
Hand-grip strength, *N*	268 (196; 326)	264 (212; 352)	244 (180; 352)	0.200	0.457	304 (280; 348)	304 (272; 370)	300 (244; 384)	0.554	0.573	**0.018**	**0.034**	**0.047**
Hand pain (0–100)	20.0 (0.0; 34.2)	0.0 (0.0; 31.6)	0.0 (0.0; 40.0)	0.260	0.272	0.0 (0.0; 0.0)	0.0 (0.0; 8.4)	0.0 (0.0; 0.0)	0.050	0.193	** < 0.001**	0.078	0.077
TST, sec	26.9 (22.1; 35.4)	23.3 18.5; 29.8)	23.2 (19.4; 30.4)	** < 0.001**	**0.001**	23.7 (21.0; 32.8)	22.3 (18.7; 26.5)	20.1 (16.8; 25.9)	** < 0.001**	** < 0.001**	0.240	0.463	**0.040**
O_2_ ml/kg × min	19.5 (15.8; 22.9)	25.0 (21.0; 31.6)	23.9 (21.6; 30.0)	** < 0.001**	** < 0.001**	18.0 (12.3; 27.7)	24.1 (19.1; 30.9)	23.0 (19.2; 27.4)	** < 0.001**	** < 0.001**	0.167	0.450	0.330
O_2_ l/min	2.0 (1.8; 2.2)	2.1 (1.7; 2.4)	2.1 (1.8; 2.6)	0.100	0.098	2,0 (1.6; 2.3)	2.0 (1.8; 2.6)	2.1 (1.7; 2.4)	**0.018**	**0.028**	0.707	0.992	0.771
SF-36 PCS (0–100)	35.8 (24.9; 46.3)	45.7 (37.0; 51.5)	46.1 (34.5; 49.8)	** < 0.001**	**0.008**	45.7 (32.9; 50.9)	52.2 (46.6; 55.7)	51.6 (43.8; 55.4)	** < 0.001**	** < 0.001**	**0.019**	**0.003**	** < 0.001**
SGPALS				**0.002**	**0.001**				**0.004**	**0.001**	0.137	0.335	0.603
PA^1^, *n* (%)	16 (39)	8 (19.5)	5 (12.8)			10 (23.3)	4 (9.3)	3 (7)					
PA^2^, *n* (%)	16 (39)	17 (41.5)	20 (51.3)			26 (60.6)	23 (53.3)	19 (48.7)					
PA^3^, *n* (%)	9 (22)	16 (39)	14 (35.9)			7 (16.3)	16 (37.2)	16 (41)					
PA^4^, *n* (%)	0	0	0			0	0	1 (2.6)					

For continuous variables, median (IQR) per participant is presented. For categorical variables, number (percentage) is presented. *TST*, timed stand test; SF-36 PCS the short form (36) health survey (physical component score; SGPALS the Saltin–Grimby physical activity level scale; *PA1*; sedentary, *PA2*, low physical activity; *PA3*, moderate physical activity; *PA4*, vigorous physical activity. Missing values at baseline: PsA group aerobic capacity: (*n* = 13), SF-36 PCS (*n* = 1); control group aerobic capacity (*n* = 8), TST (*n* = 1). Missing values at 6-month: PsA group aerobic capacity: (*n* = 13), SF-36 PCS (*n* = 6); control group aerobic capacity (*n* = 8), SF-36 PCS (*n* = 10). Missing values at 12-month PsA group aerobic capacity: (*n* = 11), SF-36 PCS (*n* = 5) control group aerobic capacity (*n* = 8), SF-36 PCS (*n* = 11)

### Muscle strength, cardiorespiratory fitness, and physical functioning within groups analysis baseline-6 months

The median TST (*p* < 0.001), SF-36PCS (*p* < 0.001) and cardiorespiratory fitness assessed with O_2_ ml/kg × min (*p* < 0.001) were significantly increased within the PsA group at 6 months compared with baseline, while cardiorespiratory fitness assessed with O_2_ l/ min was not (*p* = 0.100). The median TST, cardiorespiratory fitness assessed with O_2_ ml kg × min and O_2_ l/min, and SF-36PCS were all significantly increased (*p* < 0.001) within the control group at 6 months compared with baseline. Hand-grip strength and activity-induced hand pain remained unchanged between baseline and 6 months in both patients and controls (Table 4).

### Muscle strength, cardiorespiratory fitness, and physical functioning between groups analysis 6 months

The PsA group had still a significantly lower median peak hand-grip strength (*p* = 0.034) and median SF-36PCS (*p* = 0.019), compared with the controls. No significant difference was found for median activity-induced hand pain (*p* = 0.078), TST (*p* = 0.463), and median cardiorespiratory fitness assessed with O_2_ ml/kg × min (*p* = 0.450) and O_2_ l/min (*p* = 0.992), between the groups (Table 4).

### Muscle strength, cardiorespiratory fitness, and physical functioning within-group analysis baseline-12 months

The median TST (*p* < 0.001), SF-36PCS (*p* = 0.008), and cardiorespiratory fitness assessed with O_2_ ml/kg × min (*p* < 0.001) were significantly increased within the PsA group at 12 months compared to baseline, while median cardiorespiratory fitness assessed with O_2_ l/min (*p* = 0.098) was not. Among the controls, the median TST (*p* < 0.001), SF-36PCS (*p* < 0.001), cardiorespiratory fitness assessed with O_2_ ml/kg × min (*p* < 0.001) and O_2_ l/min (*p* = 0.028) were significantly increased at 12 months compared to baseline. No significant difference was found for median peak hand-grip strength and activity-induced hand pain in patients or controls, compared to baseline (Table 4).

### Muscle strength, cardiorespiratory fitness, and physical functioning between groups analysis 12 months

The patients had significantly lower median peak hand-grip strength (*p* = 0.047), muscle strength of the lower extremities (TST) (*p* = 0.040), and SF-36PCS (*p* < 0.001) compared to the controls. No significant differences were found for median activity-induced hand pain (*p* = 0.077) and median cardiorespiratory fitness assessed with O2 ml/kg × min (*p* = 0.330) and O2 l/ min (*p* = 0.771), between the groups (Table 4). The results of the adjusted outcome analyses between PsA and controls for age, sex, and baseline BMI were found to be nonsignificant.

## Discussion

This study aimed to evaluate the effect on physical fitness, assessed by muscle strength, cardiorespiratory fitness, body compositions, and self-reported physical functioning of a structured weight loss program, including VLED and brief support for physical activity, in patients with PsA and obesity compared to matched controls. The main findings of the study show that the intervention had positive effects on body weight, total body fat, and a negative effect on muscle mass, although the muscle strength did not deteriorate in patients with PsA. Moreover, self-reported physical functioning improved significantly in the patients while cardiorespiratory fitness remained unchanged during the study period.

A substantial weight loss was observed at 6 months for both the patients and the controls. At 12 months, the average median weight loss from baseline was 16.0% for the patients and 15.7% for the controls, implying a successful weight loss at the group level [33]. Although most diet-induced weight loss is associated with loss of fat mass, the total lean mass loss has been suggested to account for a significant part of diet-induced weight loss [34]. In the present study, the total lean mass loss from baseline was at the 12 months visit, 7% in the PsA group. The observed loss in lean mass differs from other reports with a similar intervention [35, 36]. In a study with obese women receiving VLED for 3 months followed by a 9-month weight maintenance period, the average total weight loss and lean mass loss at 12 months were 6 and 1.4 kg, respectively [36]. The relatively large difference in lean mass loss between the present study and the previous study [36] can partly be explained by baseline differences in BMI and total weight loss from inclusion at 12 months in the separate study populations.

A large weight loss accompanied by the loss of lean mass is suggested to negatively influence muscle strength [14]. In the present study, no such finding was revealed. The hand-grip strength remained unchanged during the study period, while the muscle strength in the lower extremities increased significantly in both groups. We postulate that the large weight loss among the patients in the present study lowered the mechanical load of the knee joint, possibly resulting in reduced activity-induced pain and thus better muscle function. Still, only one patient had a leg muscle strength within reference values at 6 months, while the corresponding number of patients at 12 months was five. In contrast to our findings, a previous study reported a significant reduction in leg muscle strength after 16 weeks of VLED treatment in patients with osteoarthritis and obesity [37]. The discrepancy in results for muscle strength between the present study and the previous study [37] might be due to different methods for assessments of muscle strength. The timed stand test requires both muscle strength and endurance, whereas the isometric muscle strength test assesses the absolute peak torque.

Cardiorespiratory fitness is a strong predictor of all-cause mortality and CVD morbidity in the general population [38]. Even if the cardiorespiratory fitness assessed with O_2_ ml/kg × min improved significantly in both groups during the study period, we found no such improvement for the patients when the weight factor was excluded from the analyses. Previous research has shown that greater sedentary time is associated with a higher risk of developing CVD in RA, while increased physical activity is inversely associated with CVD risk [39]. Similar findings have been reported in spondyloarthritis [40]. In the current study, 39% of the patients with PsA compared to 23% of the controls reported a sedentary lifestyle at the beginning of the study. The sedentary behavior decreased during the intervention period. At 12 months, only 13% of the patients reported a sedentary lifestyle, though only a minority (36%) of the patients reached the general recommendations for health-enhancing physical activity 1 year after the study started. Compared with previous findings [39, 40], the reduced sedentary behavior together with a significant weight loss among the patients indicates improvement in risk factors for CVD [21] despite low cardiorespiratory fitness.

Improvements in physical functioning after diet-induced weight loss has been suggested to be due to the loss of excess total body fat mass [41]. In the present study, physical functioning improved significantly in both groups over time, although the patients with PsA reported significantly lower scores on the SF-36PCS at all visits. Previous reports have shown a considerably worse physical functioning assessed with SF-36PCS in PsA compared with the general population [42]. This suggests that the physical dimension of health-related quality of life in our patients was negatively influenced not only by obesity but also by other factors related to daily activities. Increased disease activity has been suggested to lead to deterioration in muscle density resulting in poorer physical function in inflammatory arthritis [43]. While the disease activity decreased as a result of the intervention and remained low at 12 months [21], the muscle deficiency in the patients was still present. Hence, our results indicate that patients with PsA and obesity risk physical limitations in daily life due to the consequences of both their arthritis and the obesity itself.

The patients with PsA demonstrated substantial deficits in muscle strength already at baseline, which is a concern. Remarkably, only 17% of the patients compared to 67% of the controls had a grip strength corresponding to healthy reference [23] when matched for age and gender. Similar muscle deficiencies were found in the lower extremities. In fact, the muscle strength in the lower extremities for all patients was below reference values of 80 years old at baseline [24]. Excess weight and central adiposity have been suggested to exacerbate the risk of sarcopenia due to increased infiltration of fat into muscles and inflammation and insulin resistance [44]. Although sarcopenia was not the target in the present study, our findings could indicate that some of the patients were already sarcopenic at inclusion.

To counteract the loss of muscle mass during an energy restrictive diet, physical activity, especially resistance strength exercise is recommended [41]. Moreover, prescribed physical activity together with an energy-reduced diet improves physical fitness [13] and body compositions [45] compared with diet alone. In the present study, brief support from the physiotherapist, including information about the benefits of physical activity and a discussion of possible barriers and alternatives for physical activity, was given. Although the patients’ physical activity levels increased over time, muscle strength and cardiorespiratory fitness remained low in the large majority of the patients. Hence, we believe that patients with PsA and obesity undergoing weight loss treatment could benefit from more structured exercise strategies to counteract muscle mass loss and improve muscle strength and cardiorespiratory fitness.

### Strength and limitations

The main strengths of the study were the prospective design and the powerful intervention that resulted in a substantial weight loss. The clinical assessment of muscle strength, cardiorespiratory fitness, and objective assessment of body composition, in addition to self-administered questionnaires and long-term follow-up, is considered a strength. A limitation is the nonrandomized controlled design. A control group without PsA matched for age, sex, and body weight, consisting of patients with severe obesity and undergoing the same weight loss treatment program was however recruited, to enable comparisons of physical fitness. The discrepancy of the dropout rate between the groups needs to be addressed. In total, 19% of the patients and 25% of the control participants did withdraw from the study during the 12-month period. It is possible that the dropouts had poorer weight maintenance compared with the participants who continued in the study. Although, sex, age, and BMI distributions were not significantly different between the participants who withdrew or completed the study suggesting that the dropouts would have a limited impact on the results for changes in muscle strength and cardiorespiratory fitness.

## Conclusion

To conclude, a structured weight loss program of 12 months resulted in positive effects on body weight and total body fat while generating negative effects on lean body mass. Nevertheless, the muscle strength did not deteriorate in patients with PsA. This indicates that patients with PsA and concomitant obesity can benefit from weight loss treatment, including VLED, without adversely affecting muscle strength. However, the overall muscle strength and cardiorespiratory fitness were below suggested normative values for the large majority at all time points, implying that patients with PsA and obesity undergoing weight-loss treatment may benefit from more structured exercise strategies to counteract physical fitness deficiencies, thus warrant more studies.

## Data Availability

The datasets analyzed during the current study are available from the corresponding author on reasonable request.

## Abbreviations

BMI: Body mass index
CASPAR: The classification criteria for psoriatic arthritis
CRP: C-reactive protein
CVD: Cardiovascular disease
cs/b DMARD: Conventional synthetic/biologic disease modifying anti-rheumatic drug
DAPSA: The disease activity in psoriatic arthritis score
DAS28: The disease activity score of 28 joints
DXA: Dual energy x-ray absorptiometry
IQR: Interquartile range
PA: Leisure time physical activity
SGPALS: The Saltin–Grimby physical activity level scale
SF-36 PCS: The physical component scale
PsA: Psoriatic arthritis
*r*_s_: The Spearman correlation coefficient
SF-36: The short form 36
TST: The timed stand test
VAS: Visual analogue scale
VLED: Very low energy diet
